# Never a rose without a prick: pseudohyperglycemia when administering high-dose intravenous vitamin C

**DOI:** 10.1186/s13054-020-02994-4

**Published:** 2020-05-24

**Authors:** Yongli Zhang, Wei Huang

**Affiliations:** Department of Critical Care Medicine, The First Affiliated Hospital of Dalian Medical University, 222 Zhongshan Road, Dalian, 116012 LiaoNing Province China

Given its multiple biological actions, high-dose vitamin C has been considered an encouraging treatment against septic shock. Although evidence for metabolic resuscitation strategies with ascorbic acid, hydrocortisone, and thiamine is accumulating [1, 2], there are still many unresolved issues that need to be addressed. One is pseudohyperglycemia, induced by interference of ascorbic acid with point-of-care (POC) glucose measurements, which might be neglected by most physicians and easily provoke intervention with insulin and result in potential life-threatening hypoglycemia.

Briefly, in contrast to laboratory-based standard serum glucose measurement by spectrophotometric techniques using the hexokinase method, finger-stick blood glucose (FSBG) meters, or self-monitoring blood glucose (SMBG) devices, routinely utilized in ICU, rely on glucose oxidase or glucose dehydrogenase (GDH) methods [3]. The enzymatic reaction produces an electrical current, the strength of which proportionally represents the level of blood glucose. When ascorbic acid is administered, it is oxidized at the surface of the monitor, resulting in the release of an electron and negative charge. Therefore, high-dosage intravenous ascorbic acid could yield false hyperglycemia measured by POC devices, which has been documented in burn patients receiving intravenous vitamin C infusions (66 mg/kg/h) [4]. Apart from vitamin C, dopamine, acetaminophen, and icodextrin could also interfere with POC testing.

However, it needs to be mentioned that the inaccuracy of POC devices is likely to be related to plasma vitamin C concentrations. Compared with the regimen of the burn patients [4], the dose used in sepsis patients [1] was substantially lower (14 g/d for a 70-kg person), without unexpected adverse events. In addition, a retrospective study [5] suggested that most sepsis patients receiving vitamin C (9 g/d) could be managed with POC testing without major clinical impact. In the future, prospective studies should be performed to analyze the association between vitamin C dosage and plasma concentrations, the deviation of POC glucose measurements and spectrophotometric measurement, and clinical symptoms of hypoglycemia. It is also necessary to explore the impacts of concomitant confounders such as renal function and use of vasopressors, because renal dysfunction may lead to higher vitamin C plasma concentrations, and vasopressors may interfere with POC measurements.

In conclusion, when initiating high-dose vitamin C, physicians should be aware of the potential consequences of pseudohyperglycemia if employing POC devices for glucose monitoring. In this setting, the absorbance-photometric-based laboratory glucose tests rather than electrochemical detection should be used.

## Data Availability

Not applicable.

## References

[1] Fowler AA, Truwit JD, Hite RD, Morris PE, DeWilde C, Priday A, Fisher B, Thacker LR, Natarajan R, Brophy DF (2019). Effect of vitamin C infusion on organ failure and biomarkers of inflammation and vascular injury in patients with sepsis and severe acute respiratory failure: the CITRIS-ALI randomized clinical trial. JAMA..

[2] Carr AC (2019). Vitamin C administration in the critically ill: a summary of recent meta-analyses. Crit Care.

[3] Tang Z, Du X, Louie RF, Kost GJ (2000). Effects of drugs on glucose measurements with handheld glucose meters and a portable glucose analyzer. Am J Clin Pathol.

[4] Kahn SA, Lentz CW (2015). Fictitious hyperglycemia: point-of-care glucose measurement is inaccurate during high-dose vitamin C infusion for burn shock resuscitation. J Burn Care Res.

[5] Howell AP, Parrett JL. Malcom DR3. impact of high-dose intravenous vitamin C for treatment of sepsis on point-of-care blood glucose readings. J Diabetes Sci Technol. 2019. 10.1177/1932296819889638 [Epub ahead of print]. 10.1177/1932296819889638 PMC8256056 31766883

